# Diagnostic and prognostic potential of the intra-tumoral microbiota profile in HPV-independent endocervical adenocarcinoma

**DOI:** 10.3389/fcimb.2024.1440017

**Published:** 2024-08-16

**Authors:** Xin Zhou, Lili Chen, Wanrun Lin, Wenxin Zheng, Huijuan Zhang, Feng Zhou

**Affiliations:** ^1^ Departments of Pathology, The International Peace Maternal and Child Health Hospital, School of Medicine, Shanghai Jiao Tong University, Shanghai, China; ^2^ Department of Oncology, Zhejiang University School of Medicine Women’s Hospital, Hangzhou, Zhejiang, China; ^3^ Laboratory of Pathology, National Cancer Institute, National Institutes of Health, Bethesda, MD, United States; ^4^ Department of Pathology, Department of Obstetrics and Gynecology, University of Texas Southwestern Medical Center, Dallas, TX, United States; ^5^ Harold C. Simon Comprehensive Cancer Center, University of Texas Southwestern Medical Center, Dallas, TX, United States; ^6^ Shanghai Key Laboratory of Embryo Original Diseases, Shanghai, China

**Keywords:** cervical cancer, gastric-type endocervical adenocarcinoma, clear cell carcinomas, intratumoral microbiota, prognosis

## Abstract

**Background:**

Microbial community dynamics have been involved in numerous diseases, including cancer. The diversity of intertumoral microbiota in human papillomavirus independent endocervical adenocarcinoma (HPVI ECA) is not well-characterized.

**Objective:**

Our objective is to delineate the intratumoral microbiota profile in HPVI ECA and investigate its potential influence on oncogenesis.

**Methods:**

We analyzed 45 HPVI ECA cases, comprising 36 gastric-type ECA (GEA) and 9 clear cell carcinomas (CCC). We compared the microbial composition within cancerous and adjacent noncancerous tissue samples using 5R-16S ribosomal DNA sequencing. Further, we investigated the correlation between specific microbes and clinical-pathological metrics as well as patient outcomes.

**Results:**

Our findings demonstrate notable differences in the microbial spectra between cancerous and adjacent noncancerous tissues. Amongst HPVI ECA subtypes, GEAs exhibit more microbial variations compared to CCCs. Using the Random Forest algorithm, we identified two distinct microbial signatures that could act as predictive biomarkers for HPVI ECA and differentiate between GEA and CCC. Varied microbial abundances was related to clinical characteristics of HPVI ECA patients. In addition, high levels of *Micrococcus* and low levels of *unknown genus75 from the Comamonadaceae* family were associated with poorer outcomes in HPVI ECA patients. Similarly, an abundance of *Microbacterium* correlated with reduced overall survival (OS), and a high presence of *Streptococcaceae* family microbes was linked to reduced recurrence-free survival (RFS) in GEA patients. Intriguingly, a high abundance of *Micrococcus* was also associated with a worse OS in GEA patients.

**Conclusion:**

The study reveals distinct microbial signatures in HPVI ECA, which have potential as biomarkers for disease prognosis. The correlation between these tumor-associated microbiota features and clinicopathological characteristics underscores the possibility of microbiome-based interventions. Our research provides a foundation for more in-depth studies into the cervical microbiome’s role in HPVI ECA.

## Introduction

Endocervical adenocarcinoma (ECA) is the second most common histological subtype of cervical cancer, accounting for approximately 20-25% of all cases in Western countries (Bergström et al., 1999; Smith et al., 2000; Adegoke et al., 2012). While the majority of cervical squamous cell carcinomas (SCC) are associated with human papillomavirus (HPV) infection, a notable 10-25% of ECAs are HPV-independent (HPVI) (Kusanagi et al., 2010; Karamurzin et al., 2015; Stolnicu et al., 2018). The 2020 World Health Organization (WHO) classification of tumors of the female reproductive organs includes several subtypes of HPVI category, namely gastric-type ECA (GEA), clear cell carcinoma (CCC), mesonephric carcinoma, endometrioid carcinoma and adenocarcinoma not otherwise specified (Parra-Herran et al, 2020). Among these, GEA is the most prevalent subtype of HPVI ECA, representing 10-15% of all ECAs globally and up to 25% in Asian populations (Kusanagi et al., 2010; Karamurzin et al., 2015). CCC is another subtype of HPVI ECA, constituting 4-9% of all ECA cases (Noller et al., 1974; Saigo et al., 1986).

HPVI ECAs often present with more aggressive behavior, a propensity for extrauterine spread, and are frequently diagnosed at an advanced-stage, leading to a less favorable prognosis when compared to HPV associated ECAs (Kojima et al., 2007; Karamurzin et al., 2015; Nishio et al., 2019). The critical role of HPV in most cervical adenocarcinomas notwithstanding, it is insufficient to explain the etiology and progression of HPVI ECA alone. This gap in understanding underscores the urgent need for research into additional factors that may influence the onset and advancement of this cancer type.

Emerging evidence posits the microbiota as a pivotal regulatory agent and potential biomarker in the pathogenesis of various cancers, including that of the cervix (Meng et al., 2018; Liu et al., 2019a; Wu et al., 2021). Imbalances in microbial community homeostasis can trigger cancer initiation and growth through a variety of complex processes. These include the induction of inflammation, impairment of immune function, toxin production, DNA damage, activation of oncogenic pathways, and alteration of anticancer drug pharmacokinetics (García-Castillo et al., 2016; Byrd et al., 2023). Furthermore, the extensive microbiota, considered a secondary genome within the host, is instrumental in molding the tumor microenvironment and thus impacts both cancer progression and the efficacy of treatment (Pushalkar et al., 2018; Jain et al., 2021).

Current research has established links between cervical/vaginal microbiota and cervical lesions (Mitra et al., 2015; Audirac-Chalifour et al., 2016; Wu et al., 2021; Lin et al., 2022; Jiang et al., 2023; Teka et al., 2023; Wu et al., 2023), but the specific microbial species or communities associated with ECA incidence, clinical pathological features, and prognosis remain largely unexplored, particularly for HPVI ECA. This study, aims to delineate the differences in microbial abundance between cancerous and adjacent noncancerous tissues and further investigate the intratumoral microbiota’s role in cancer prediction and subtype differentiation. We also investigate the associations between microbiota profile and clinical-pathological variables, as well as prognostic implications for the disease.

## Method and material

### Sample collection

Paraffin-embedded samples from 45 HPVI ECA cases (36 GEA and 9 CCC) from March 2018 to February 2023. Patients’ clinicopathologic information, including age, clinical stage, and follow-up data were extracted from the electronic clinical information system database of Women’s Hospital. Hematoxylin and eosin and immunohistochemistry (IHC) slides were reviewed by two gynecological pathologists (X.Z. and F.Z.) in a blinded fashion and the pathologic diagnosis was confirmed. The sample collection procedure and further studies were approved by the Institutional Review Board and Ethics Committee of the Zhejiang University School of Medicine Women’s Hospital, China (IRB-20210276R).

Subsequently, 5R-16S ribosomal DNA (5R-16S rDNA) MiSeq sequencing was performed on these samples. All experiments were conducted following the guidelines and instructions approved by the manufacturer. Clinical staging of patients was categorized according to the staging system for gynecologic tumors developed by the International Federation of Gynecology and Obstetrics (FIGO) (Bhatla et al., 2018).

### Human papillomavirus RNA *in situ* hybridization

High-risk HPV (hrHPV) testing was performed using RNA *in situ* hybridization (RISH) (Advanced Cell Diagnostics, Hayward, CA, USA), which recognizes 18 hrHPV genotypes (16,18, 26, 31, 33, 35, 39, 45, 51, 52, 53, 56, 58, 59, 66, 68, 73, and 82) according to the manufacturer’s instructions. The presence of intracellular punctate yellow-to-brownish reaction was defined as positive staining for HPV RNA (Wang et al., 2023).

### DNA extraction and 5R-16S rDNA sequencing

DNA from various samples was extracted using the formalin-fixed and paraffin embedded (FFPE) Genome DNA Extraction Kit (Concert, Xiamen, China) following the manufacturer’s guidelines. The integrity and fragment size of the extracted DNA were assessed through 1% agarose gel electrophoresis. Additionally, the quality of the extracted DNA was measured using NanoDrop 2000 (Thermo Scientific, Waltham, MA, USA).

Microbial community analysis was performed using 5R-16S rDNA sequencing technology (Lianchuan Biotechnology, Hangzhou, China). To amplify the five regions (V2, V3, V5, V6, and V8) on the 16S rDNA, specific primers were utilized in two rounds of amplification. Primers and amplification procedures were applied as described in previous reference (Nejman et al., 2020). Following amplification, the PCR products underwent purification using AMPure XT beads (Beckman Coulter Genomics, Danvers, MA, USA) and quantification via Qubit (Invitrogen, USA). Subsequently, amplicon pools were readied for sequencing, with the size and quantity of the amplicon library evaluated on an Agilent 2100 Bioanalyzer (Agilent, USA) and using the Library Quantification Kit for Illumina (Kapa Biosciences, Woburn, MA, USA), respectively. Following sequencing of the libraries on the NovaSeq 6000 platform, a total of 18,814,031 reads were obtained after quality control and filtering, with an average of 209,045 reads per sample.

### Microbiome data analysis

Paired-end reads were sorted into samples using unique barcodes and then trimmed by removing the barcode and primer sequences using cutadapt (v1.9). FLASH (v1.2.8) was employed for merging paired-end reads. Quality filtering of raw reads was performed with fqtrim (v0.94) under specific filter conditions to obtain high-quality clean reads. Chimeric sequences were filtered using Vsearch software (v2.3.4). Following dereplication of reads and discarding of singletons using DADA2, the representative sequence with single-base accuracy is obtained, resulting in the ASV (Amplicon Sequence Variants) feature table and feature sequence. The ASV feature sequence alignment for species annotation was performed using BLAST, with the SILVA and NT-16S alignment databases. Subsequently, species abundance per sample was calculated based on the ASV abundance table. Annotation confidence was set at a threshold of 0.7.

The α diversity indexes (observed species, Chao1, Shannon, Simpson) was implemented to display the diversity of bacteria among the different samples. The β diversity analysis was used to evaluate global differences between microbial community composition. Bray-Curtis for Nonmetric multidimensional scaling (NMDS) was carried out to visualize the β diversity between different groups. The α diversity and β diversity were calculated by QIIME, relative abundance was used in bacteria taxonomy. Linear discriminant analysis effect size (LEfSe) was employed to assess differences in abundance of taxonomic units between different groups (Segata et al., 2011), with those having linear discriminant analysis (LDA) values greater than 3 and *p*-values less than 0.05 considered to have significant differential abundance. Wilcoxon rank-sum tests were employed for statistical comparisons of taxonomic units at the phylum and genus levels between different groups. Additionally, using Phylogenetic Investigation of Communities by Reconstruction of Unobserved States2 (PICRUSt2) based on the Kyoto Encyclopedia of Genes and Genomes (KEGG) database and Cluster of Ortholog Genes (COG) database (Douglas et al., 2020), the functional composition of bacterial communities between different groups was predicted using STAMP v2.1.3 (Parks et al., 2014). A *p*-value less than 0.05 was considered statistically significant.

### Microbiota signature identification

In this study, we employed the Random Forest (RF) algorithm for the selection of microbiota signatures. RF is a popular supervised machine learning method used for modeling various omics data, including microbiome data, and it is highly effective for both prediction and interpretation purposes. A classification model was established using the RF algorithm from the *RandomForest* R package (https://cran.r-project.org/web/packages/randomForest/index.html). The receiver operating characteristic (ROC) curve was constructed using the *pROC* R package (https://cran.r-project.org/web/packages/pROC/index.html), and the performance of the prediction model was evaluated through the area under the ROC curve (AUC).

### Statistics analysis

Data analysis was performed using GraphPad Prism and R. Statistical tests were based on two-sided comparisons, with significance set at p < 0.05. When stratifying according to clinical pathological features, the Wilcoxon test was employed to determine differential enrichment of bacterial taxa. The Pearson *“cor.test ()* “ function in R version 4.0.2 was utilized to analyze the correlation between specific microbial relative abundance and tumor staging. Consistent with our previous studies (Sun et al., 2024), overall survival (OS) was defined as the time from surgery to death or the end of follow-up, while recurrence-free survival (RFS) was defined as the interval between total hysterectomy and first recurrence, metastasis or last observation. Following this, samples were classified into high-abundance and low-abundance groups based on the microbial composition abundance. Kaplan‐Meier survival analysis was conducted to assess microbial communities associated with RFS and OS.

## Result

### Clinicopathologic characteristics of patients

**Figure 1:**
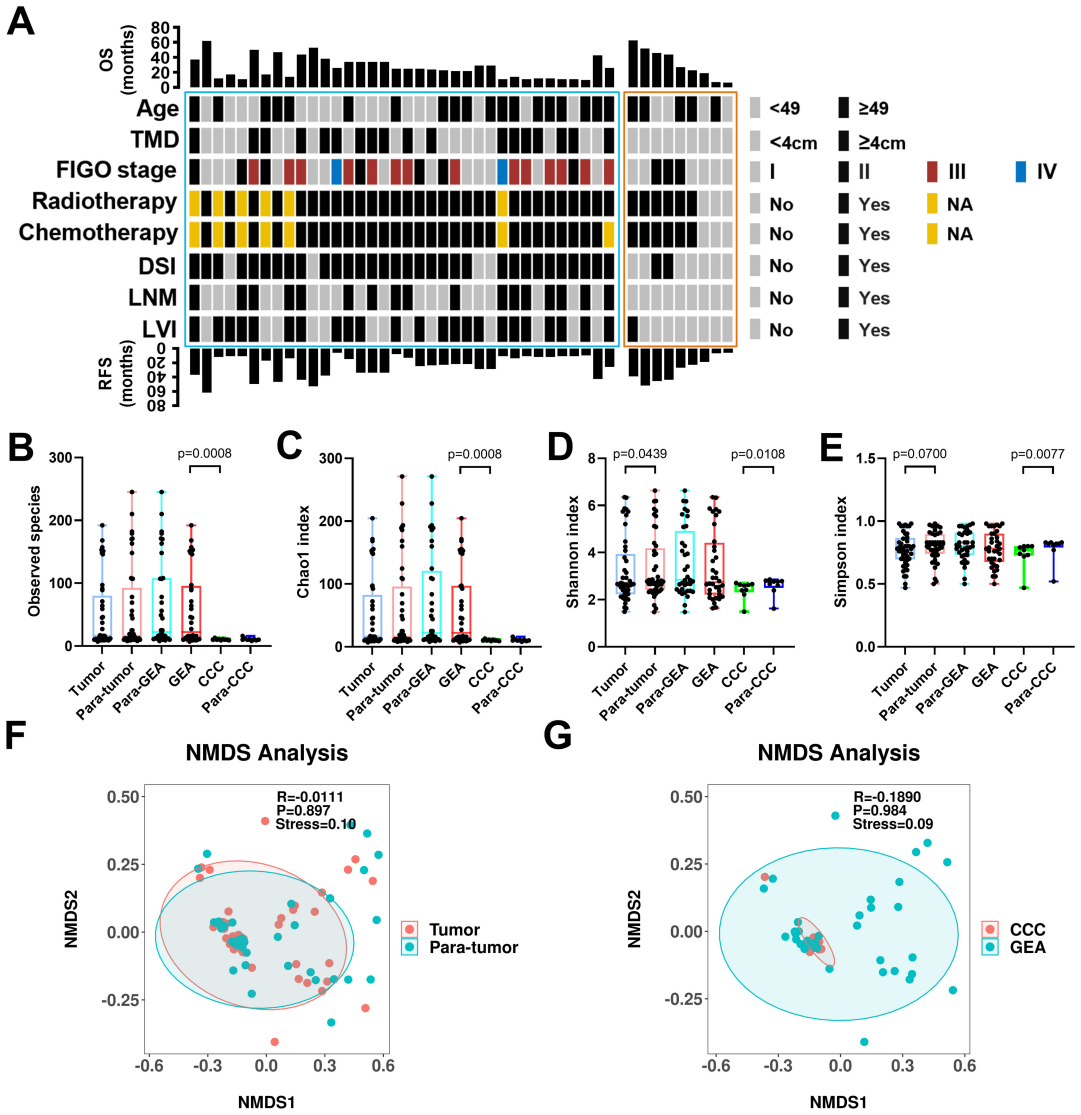
Clinical-pathological characteristics and intra-tumoral microbiota diversity of all patients. **(A)** Clinical-pathological characteristics and clinical outcomes of all 45 human papillomavirus independent endocervical adenocarcinoma (HPVI ECA) patients. Cases in the blue box belong to gastric-type endocervical adenocarcinoma (GEA) patients, and cases in the red box belong to clear cell carcinoma (CCC) patients. **(B-E)** The α-diversity among these groups was evaluated using the two-sided Wilcoxon sum rank test for the following indices: Box plots of observed species **(B)**, Chao1 index **(C)**, Shannon index **(D)**, and Simpson index **(E)** in HPVI ECA samples. **(F, G)** β-diversity assessed by non-metric multidimensional scaling (NMDS) using Bray-Curtis. **(F)** HPVI ECA cancerous tissue versus adjacent non-cancerous tissue, **(G)** GEA versus CCC. TMD, tumor maximum diameter; DSI, deep stromal invasion; LNM, lymph node metastasis; LVI, lymphovascular invasion.

This study included 45 patients with HPVI ECA, with 36 patients with GEA and 9 with CCC. The median age of these patients was 49 years (49 years for the GEA and 58 years for the CCC). The median follow-up duration for the cohort was 27 months with GEA patients having a median of 26 months and CCC patients 32 months. Details on patient demographics, pathologic diagnoses, clinical stage (FIGO stage), tumor maximum diameter (TMD), deep stromal invasion, lymph node metastasis (LNM), and lymphovascular invasion (LVI), and follow-up data are summarized in **Table 1** and **Figure 1A**.

**Table 1:** Clinicopathological information of patients.

Clinicopathological parameter	HPVI ECA		GEA		CCC	
	N	%	n	%	n	%
Median Age (range)	49 (19-76)		49 (33-76)		58 (19-66)	
< Median Age	21	46.7%	17	47.2%	4	44.4%
≥ Median Age	24	53.3%	19	52.8%	5	55.6%
Stage (FIGO)						
I	19	42.2%	13	36.1%	6	66.7%
II	10	22.2%	7	19.4%	3	33.3%
III	14	31.1%	14	38.9%	0	0
IV	2	4.4%	2	5.6%	0	0
Tumor Maximum Diameter						
< 4 cm	27	60%	18	50%	9	100%
≥ 4 cm	18	40%	18	50%	0	0
Deep Stromal Invasion						
Yes	34	75.6%	32	88.9%	2	22.2%
No	11	24.4%	4	11.1%	7	77.8%
Lymphovascular Invasion						
Yes	24	53.3%	23	63.9%	1	11.1%
No	21	46.7%	13	36.1%	8	88.9
Lymph Node Metastasis						
Yes	17	37.8%	17	47.2%	0	0
No	28	62.2%	19	52.8%	9	100%
Radiotherapy/chemotherapy						
Yes	36	80%	30	83.3%	6	66.7%
NO	3	6.7%	0	0	3	33.3%
NA	6	13.3	6	16.7%	0	0

### Intratumor microbial diversity in HPVI ECA

We compared the microbial communities within cancerous lesions (tumor group) to those in the adjacent non-cancerous tissues (para-tumor group) across the 45 HPVI ECA patients. We also analyzed the differences between the GEA and CCC groups. In our result, no significant differences in the Observed species were noted between the tumor and para-tumor groups. However, species richness was significantly greater in the GEA than in the CCC (Observed species, *p* = 0.0008) (**Figure 1B**). The Chao1 species richness index also confirmed this significant difference (Chao1 *p* = 0.0008) (**Figure 1C**). Marginally significant variations were seen in microbial community α-diversity between cancerous and adjacent non-cancerous tissues (Shannon *p* = 0.0438, Simpson *p* = 0.0700). However, between CCC and its adjacent tissue, the α-diversity shows a significant difference (Shannon *p* = 0.0108, Simpson *p* = 0.0077) (**Figures 1D, E**). Regarding β-diversity, Bray-Curtis dissimilarity assessments and NMDS plots revealed no significant difference between the tumor and para-tumor groups or between the GEA and CCC groups, though a certain explanatory meaning observed in these grouping scheme (0.05 < stress < 0.1), as shown in **Figures 1F, G**.

### Altered composition of the intratumor microbiome in HPVI ECA

**Figure 2:**
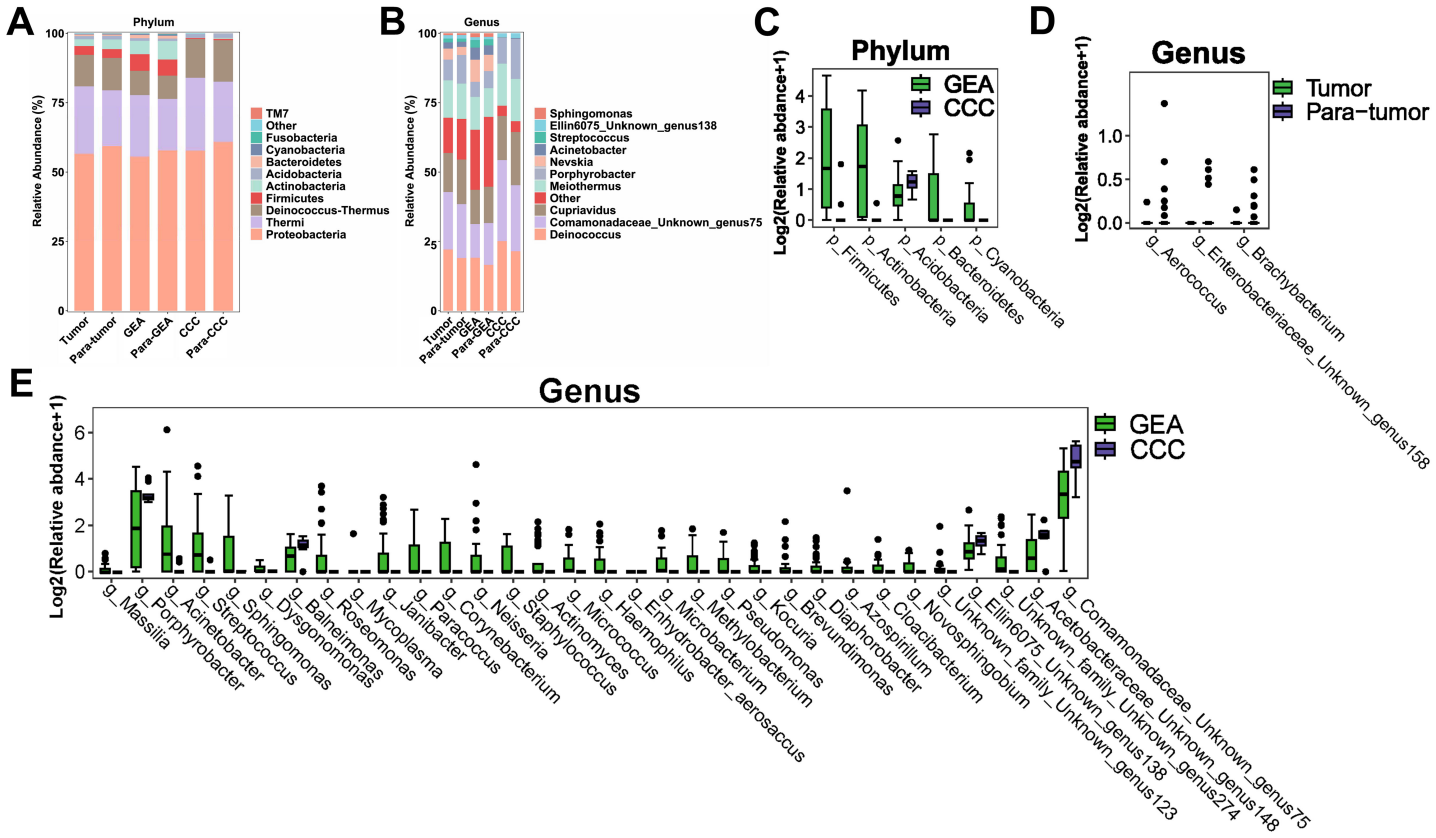
Microbial composition and differences between cancerous lesions and adjacent tissues. **(A)** Stacked bar chart showing the average relative abundance of the top 10 phyla across all groups. **(B)** Stacked bar chart depicting the average relative abundance of the top 10 genera across all groups. **(C-E)** Boxplots evaluating the statistical differences of intra-tumoral microbiota at the phylum and genus levels. **(C)** Genera with differential abundance between Tumor and Para-tumor groups. **(D)** Phyla with differential abundance between gastric-type endocervical adenocarcinoma (GEA) and clear cell carcinoma (CCC) groups. **(E)** Genera with differential abundance between GEA and CCC groups.

At the phylum level, *Proteobacteria, Thermi*, and *Deinococcus-thermus* were predominantly found across all groups, as shown in **Figure 2A**. A more detailed examination at the genus level highlighted a significant enrichment of *Deinococcus*, as well as *Unknown genus75 of family Comamonadaceae*, *Cupriavidus*, *Meiothermus*, and *Porphyrobacter* across all groups. Notably, *Nevskia* was markedly more abundant in the GEA and its adjacent non-cancerous groups, as presented in **Figure 2B**. The average abundance ratios of the top 10 microbial compositions at the phylum and genus levels for each group are presented in **Supplementary Table S1**. Microbial community compositions at other taxonomic levels are detailed in **Supplementary Figure S1**.

Comparative analysis using the Wilcoxon rank-sum test revealed no significant differences in microbial composition at the phylum level between the HPVI ECA and adjacent non-cancerous groups. However, when comparing GEA and CCC groups, relative abundances of *Actinobacteria, Firmicutes, Betaproteobacteria, Bacteroidetes, Cyanobacteria*, and *Acidobacteria* were significantly different (**Figure 2C**). At the genus level, differences in the microbial composition of *Brachybacterium, Unknown genus158 of family Enterobacteriaceae*, and *Aerococcus* were statistically significant within cancer tissues compared to paired adjacent tissues, as indicated in **Figure 2D**. Box plots in **Figure 2E** showed 32 microbial genera were significantly different between the GEA and CCC. Taxonomic differences at the phylum and genus levels and their corresponding *p*-values are detailed in **Supplementary Table S2**.

**Figure 3:**
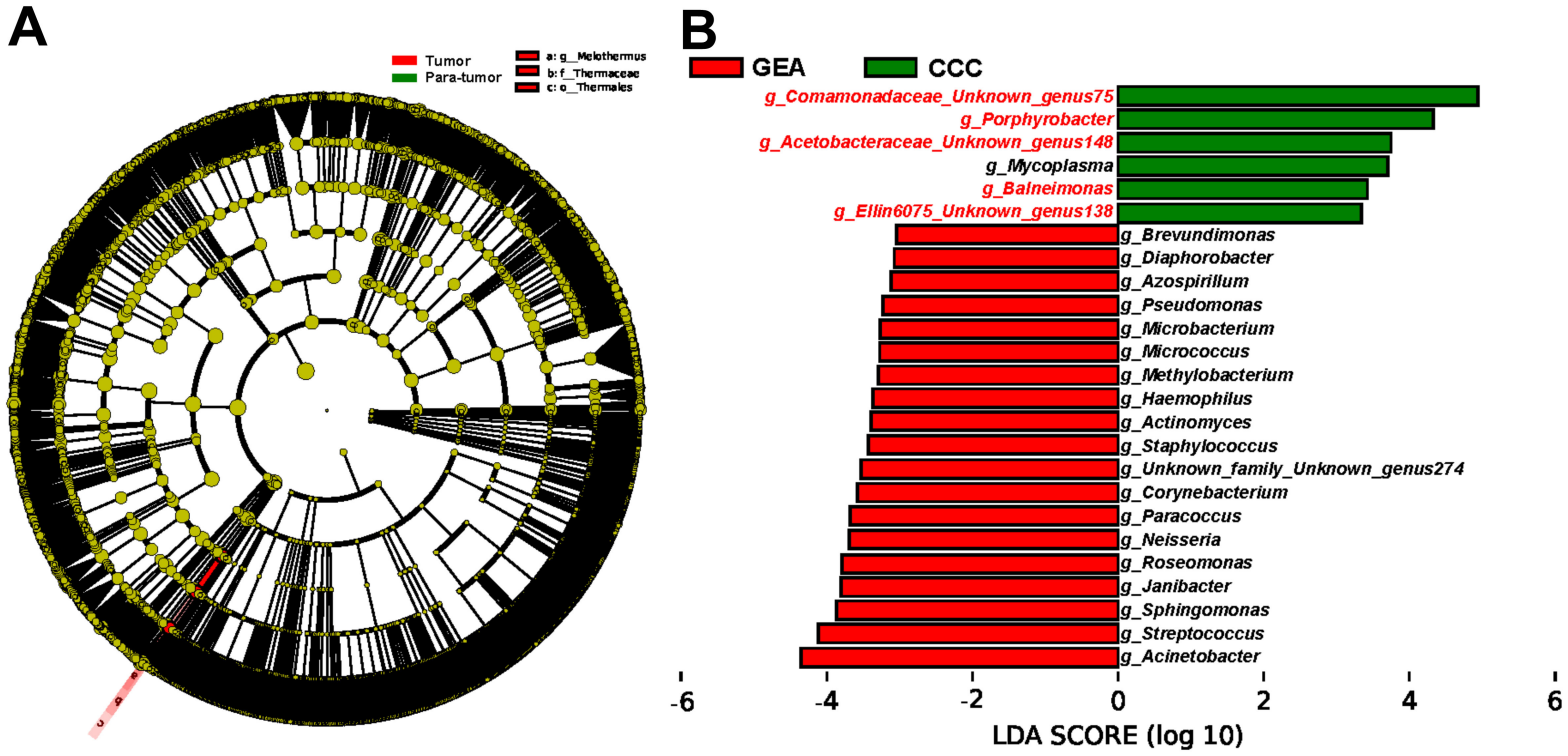
Identification of enriched microbial taxa between groups using Linear Discriminant Analysis Effect Size (LEfSe) method. **(A)** Cladogram illustrating taxonomic enrichment of microbial taxa between cancerous lesion and adjacent tissues. **(B)** Linear Discriminant Analysis (LDA) scores of specific microbial taxa at the genus level between gastric-type endocervical adenocarcinoma (GEA) and clear cell carcinoma (CCC) groups. Genera highlighted in red font indicate taxa overlapping with microbes in the subsequent Random Forest (RF) model distinguishing GEA from CCC, with an LDA score threshold of 3.

Furthermore, LEfSe was utilized to corroborate these statistical differences and to identify significant taxonomic groups within the various microbial communities. A substantial increase in the relative abundance of *Teiothermus, Thermaceae*, and *Thermales* was observed in cancerous lesions, as shown in **Figure 3A**. At the genus level, LEfSe results identified 25 microbial genera with significant differences between GEA and CCC (**Figure 3B**). Additionally, we found that among all taxonomic categories, 77 bacteria had increased relative abundance in the GEA, while 22 bacteria were more abundant in CCC, detailed in **Supplementary Figure S2**.

### The role of intratumoral microbiota in HPVI ECA prediction and subtype differentiation

**Figure 4:**
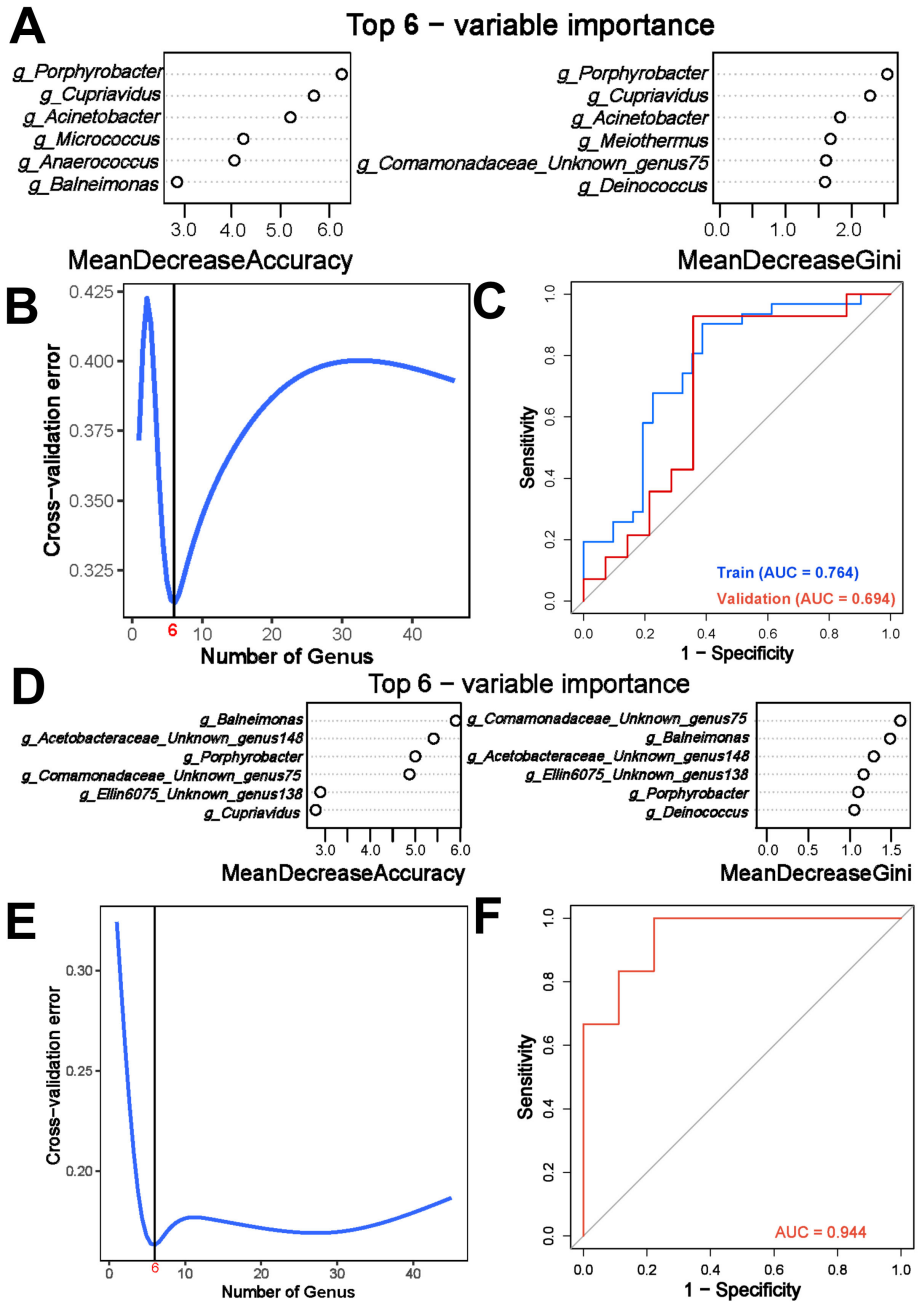
Utilization of Random Forest (RF) algorithm for tumor prediction and discerning tumor subtypes based on intratumoral microbiota signature. **(A)** Prioritization of the top 6 genera between human papillomavirus independent endocervical adenocarcinoma (HPVI ECA) tissues and adjacent tissues using 10-fold cross-validation method. **(B)** Cross-validation error curves between cancerous and adjacent non-cancerous tissues demonstrating the 10-fold cross-validation method employed to determine prioritization of top features for constructing simplified models. **(C)** The Receiver Operating Characteristic (ROC) curve illustrating predictive performance of simplified RF model constructed based on the top 6 genera. Blue and red curves represent model performance in training set (31 pairs) and validation set (14 pairs) respectively. **(D)** Prioritization of the top 6 genera between gastric-type endocervical adenocarcinoma (GEA) and clear cell carcinoma (CCC) using 10-fold cross-validation method. **(E)** Cross-validation error curves between GEA and CCC illustrating the 10-fold cross-validation method employed to determine prioritization of top features for constructing simplified models. **(F)** ROC curve illustrating predictive performance of simplified RF model for distinguishing between GEA and CCC based on the top 6 genera.

Exploration of microbial signatures within tumor micro environments has now made it possible to identify potential diagnostic markers for HPVI ECA. In general terms, a “microbial signature” refers to a collection of microbial taxa that are closely associated with a specific trait and have a high predictive value within a given model (Rivera-Pinto et al., 2018). The RF method, a machine learning algorithm, excels at identifying an optimal set of variables with significant discriminative power from a large pool of dependent or independent variables. It is renowned for its capability to capture complex dependency patterns between the outcome and the covariates (Hornung and Wright, 2019). A training cohort consisting of 31 pairs of HPVI ECA cancerous and adjacent tissue samples was used to develop a RF prediction model. And the top six genera were selected through a 10-fold cross-validation (**Figures 4A, B**). The detailed genera composition in each sample was listed in **Supplementary Table S3**. The model’s effectiveness was confirmed not only in the training cohort but also in a validation cohort of 14 pairs of HPVI ECA samples, achieving AUC scores of 0.764 and 0.694 for the training and validation sets, respectively (**Figure 4C**).

Additionally, LEfSe analysis highlighted significant alterations between microbial profiles of GEA and CCC groups, underscoring the importance of microbial signatures in differentiating these conditions. To ensure an adequate sample size, all samples of GEA and CCC were included in the analysis. Adhering to the principle of simplicity, the top 6 genera were then selected for the final model (**Figures 4D, E**), which exhibited outstanding predictive accuracy (AUC = 0.944) as shown in **Figure 4F**. When constructing these models, a taxon was included only if it was present in over 20% samples.

### Microbial abundances and their clinical implications

**Figure 5:**
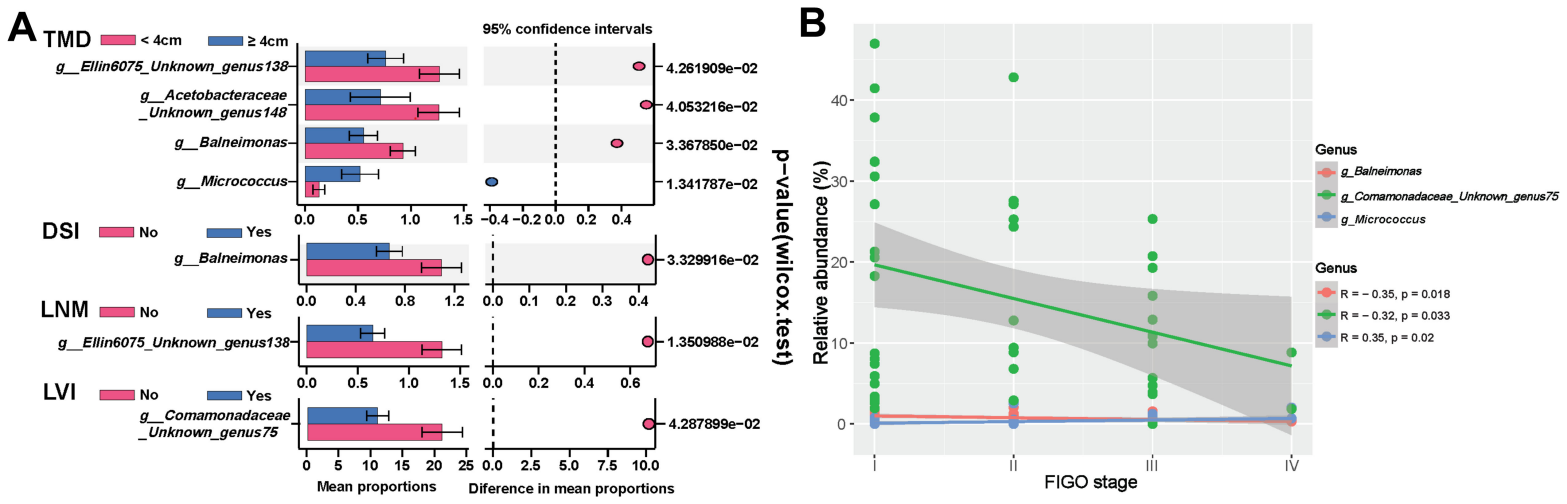
Correlation between intratumoral microbiota and clinical-pathological features of human papillomavirus independent endocervical adenocarcinoma (HPVI ECA). **(A)** Differential enrichment of specific microbiota across stratifications based on distinct clinical characteristics. **(B)** Relationship between specific microbiota and tumor stage. TMD, tumor maximum diameter; DSI, deep stromal invasion; LNM, lymph node metastasis; LVI, lymphovascular invasion.

Further investigation into the correlation between specific microbes and clinical characteristics of HPVI ECA patients revealed varied microbial abundances. Patients with LNM showed a notably lower presence of *Unknown genus138 of family Ellin6075* (*p*=0.0135). *Balneimonas* exhibited significantly lower abundance in patients with deep stromal invasion (*p*=0.0333). Notably, the low-abundance of *Unknown genus75 of family Comamonadaceae* was also linked to LVI (*p*=0.0429). Additionally, patients with larger tumors (≥ 4cm) exhibited a decrease in *Balneimonas* (*p*=0.0336), *Unknown genus138 of family Ellin6075* (*p*=0.0426), *Unknown genus148 of Acetobacteraceae* (*p*=0.0405), and an increase in *Micrococcus* (*p*=0.0134) (**Figure 5A**). Advanced tumor stages correlated significantly with the abundance of certain microbes: *Balneimonas* (*p*=0.018, r=-0.35), *Unknown genus75 of family Comamonadaceae* (*p*=0.033, r=-0.32) were inversely related to advanced tumor stage, while *Micrococcus* exhibited a positive correlation (*p*=0.02, r=0.35) (**Figure 5B**).

**Figure 6:**
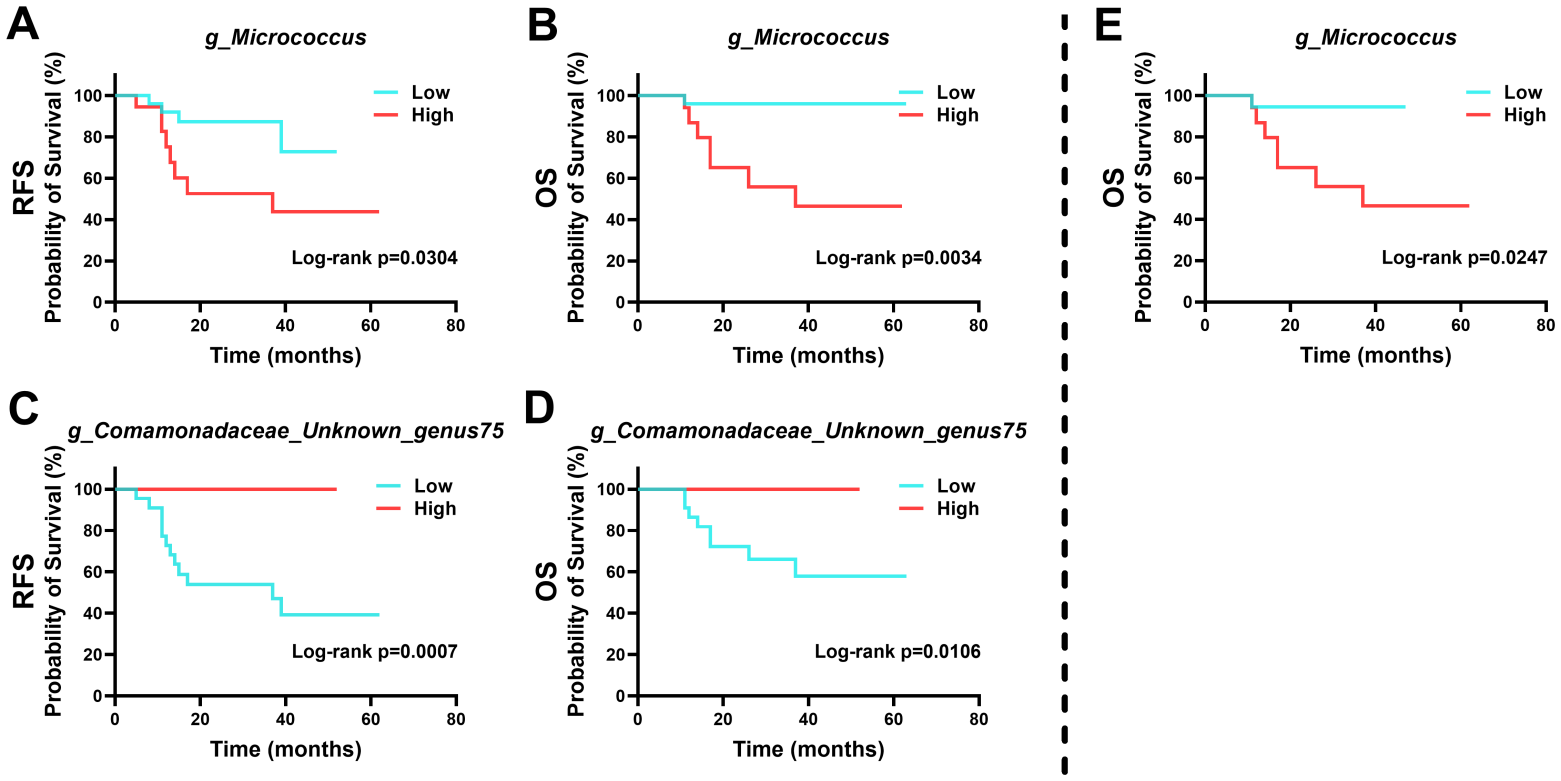
Kaplan-Meier survival curves based on intra-tumoral microbial abundance. **(A-D)** Relationship between intra-tumoral microbiota and prognosis of human papillomavirus independent endocervical adenocarcinoma (HPVI ECA) patients. **(E)** Relationship between intra-tumoral microbiota and prognosis of gastric-type endocervical adenocarcinoma (GEA) patients.

Survival analysis indicated that specific microbial abundances could be indicative of recurrence and survival probabilities in patients (**Figure 6**). High levels of *Micrococcus* and low levels of *Unknown genus75 of family Comamonadaceae* were associated with poorer outcomes in HPVI ECA patients. Intriguingly, a high abundance of *Micrococcus* was also associated with worse OS in GEA patients.

### Predicted functional impact of the HPVI ECA microbiome

**Figure 7:**
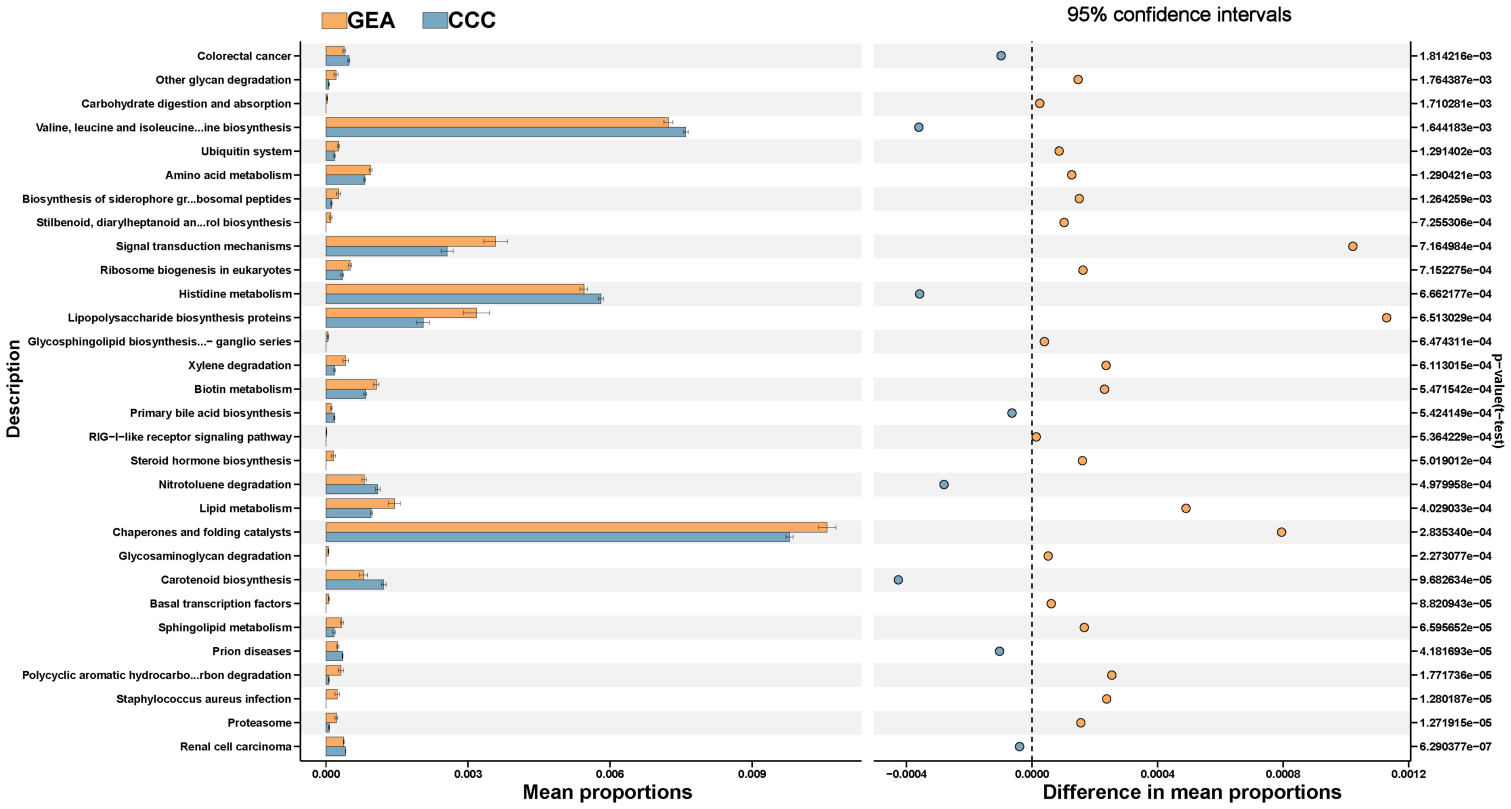
The top 30 Kyoto Encyclopedia of Genes and Genomes (KEGG) pathways between gastric-type endocervical adenocarcinoma (GEA) and clear cell carcinoma (CCC) groups, along with their respective 95% confidence intervals and *p*-values.

The study annotated and analyzed KEGG pathways between the GEA and CCC groups, uncovering 106 pathways with significant variances (**Supplementary Table S4**). The GEA group exhibited an increase in 64 pathways including those involved in proteasome function, polycyclic aromatic hydrocarbon degradation, and various metabolic processes, while the CCC group showed enrichment in 42 pathways, including those related to sphingolipid metabolism and biosynthesis of certain amino acids and bile acids (**Figure 7**). These data suggest a significant metabolic reprogramming within the microbial communities in the HPVI ECA subtype. However, when comparing HPVI ECA tumors to adjacent tissues, no significant changes were noted in KEGG pathways, indicating a microbial similarity between these groups. Yet, nine COG functional categories differed significantly between the two groups (**Supplementary Figure S3**, **Supplementary Table S5**).

## Discussion

Mounting evidence increasingly implicates dysbiosis of the reproductive tract microbiota as a key risk factor for the onset of cervical cancer (Hu et al., 2018; Zhou et al., 2022). Despite a rising incidence of cervical adenocarcinoma (Smith et al., 2000), no studies have reported on microbial dysbiosis related to the development of this disease in patients. In our research, we concentrated on tissue samples from HPVI ECA to evaluate both the composition and the variation of the microbiota, alongside its prognostic implications. Our principal discoveries are as follows: 1) microbial profiles in HPVI ECA exhibit alterations, displaying slightly deviations from neighboring tissues; 2) amongst HPVI ECA subtypes, GEA reveals more pronounced microbial profiles changes compared to CCC; 3) We employed the RF algorithm to reveal distinct microbial signatures for predicting HPV-induced ECA carcinogenesis composed of seven bacterial species, as well as another microbial signature discriminating between HPVI ECA subtypes (GEA and CCC) involving six bacterial species; 4) We identified specific bacterial profiles that correlated with both the prognosis and the clinical-pathological features of HPVI ECA.

By employing 5R-16S rDNA sequencing, we delineated the microbial composition of 45 matched pairs of HPVI ECA carcinomas and their adjacent non-cancerous tissue. The disparity in microbial species abundance and richness between cancerous and adjacent non-cancerous tissues was negligible in terms of both α-diversity and β-diversity, which may be due to their physical closeness. However, in GEA, microbial abundance was significantly greater than in CCC. Literature suggests that a diminished microbial community diversity might correlate with disease progression, while a higher diversity typically signals a healthier condition, as noted in studies on gastric cancer (Liu et al., 2019b) and pancreatic cancer (Riquelme et al., 2019). Similar to these findings, our study discovered significantly lower microbial α-diversity (Shannon *p =* 0.0439) indices between HPV-associated endocervical adenocarcinoma (ECA) carcinogenic lesions and adjacent tissues, indicating a decrease in diversity associated with cancer. These patterns suggest that the disease’s advancement could be related to either a simplification or a diversification of microbial communities within the ecosystem of the microbiota.

Within the scope of our investigation, the predominant phylum in both the cancerous and adjacent tissues was *Proteobacteria*, aligning with previous findings on the microbiota in cervical cancer (Chang et al., 2023a; Mulato-Briones et al., 2024). Additionally, the relative abundance of *Thermi* and *Deinococcus-thermus* exceeded 10%, yet did not display significant variances between the groups. Notably, although microbes such as *Firmicutes and Actinobacteria* were reported as dominant in earlier studies (Chang et al., 2023a; Mulato-Briones et al., 2024), they did not appear as prevalent in our research, possibly due to the distinct nature of our samples or specific microbial traits linked to HPVI ECA. Importantly, we observed variations in the relative abundance of these microbes between GEA and CCC, hinting at their nuanced involvement in the evolution of HPVI ECA subtypes. At the genus level, the microbial community within HPVI ECA cancerous tissues, as opposed to adjacent non-cancerous tissues, was predominantly composed of *Deinococcus, Unknown genus75 of family Comamonadaceae, Cupriavidus, Meiothermus, Porphyrobacter*. Notably, *Meiothermus* as identified in further LEfSe analysis, was more prevalent in cancerous lesions, suggesting its potential as a biomarker for the onset and progression of HPVI ECA.

The RF algorithm has proven to be an effective method for predicting different disease states, particularly through the identification of microbial signatures (Yu et al., 2017; Nakatsu et al., 2018; Half et al., 2019; Yang et al., 2023). This allows researchers to delve into the complexities of microbial dysbiosis and identify targets for managing these imbalances. Prior research by Wu et al. (Wu et al., 2023) and Chang et al (Chang et al., 2023b). has applied the RF algorithm to identify distinctive microbial features in patients with cervical cancer, and to correlate these with tumor stages and prognostic outcomes. Additionally, Jiang et al. have constructed a RF-based risk model aimed at predicting the metastasis of cervical cancer (Jiang et al., 2023). Notably, the two RF models established in our study both demonstrate high predictive accuracy. Furthermore, there were significant differences in the abundance of these bacteria between the two groups. The highly consistent results among these analyses explain the remarkable ability of this group of bacteria to distinguish between the two HPVI ECA subtypes. Out of the ten bacterial markers identified through the two RF models, *Anaerococcus* (Chen et al., 2020) and *Porphyrobacter* (Lin et al., 2022) has been demonstrated a direct association with cervical intraepithelial neoplasia. Moreover, *Micrococcus*, which is implicated in inflammatory responses in endometrial cancer, has been linked to a higher incidence of cervical cancer (Lu et al., 2021), and its increased abundance is associated with cervical cancer (Tango et al., 2020).

We further explored the relationship between the abundance of certain microbe and the clinical outcomes of patients. Notably, a higher abundance of *Micrococcus* has been linked with worse prognosis in patients with HPVI ECA, and is positively correlated with the advanced clinical stage, indicating its potential role in the progression of cancer. The discovery of microbial markers such as *Balneimonas*, *Unknown genus75 of family Comamonadaceae* and *Unknown genus138 of family Ellin6075* could provide valuable insights into the mechanisms underlying tumor metastasis. The development of agents targeting these bacteria and their associated derivatives holds tremendous promise in cancer therapy. Various bacterial derivatives have been reported to selectively target cancer cells and exert cytotoxic effects, including bacterial toxins, enzymes, peptides, and some secondary bacterial metabolites. Current research has identified several *Micrococcus* with anticancer activity, such as the ethyl acetate extract and yellow pigment fro*m Micrococcus luteus, w*hich exhibit anticancer effects against cancer cells (Mithun and Rao, 2012; Ushasri and Shalomi, 2015). Other reports indicate that the carotenoids pigment from *Micrococcus roseus* possess antioxidant and anticancer properties (Rostami et al., 2016). Furthermore, a recent study revealed that the yellow pigment from *Micrococcus terreus* can activate cysteine to induce apoptosis in cervical cancer cell lines (Shukla and Nadumane, 2021). This burgeoning field of research underscores the potential of bacterial derivatives as valuable additions to the arsenal of cancer therapeutics, warranting further exploration and clinical translation.

The research presented here offers a preliminary exploration of the microbial community within cervical adenocarcinoma tumors. However, it is not without limitations, particularly the potential for the 16S rDNA amplification and sequencing approach to overlook certain members of the microbial community. This technique may not capture as comprehensive a picture as metagenomic sequencing and leaves some microbial taxa unidentified. Additionally, the limited sample size and lack of diversity in the tissue samples may constrain the wider applicability of these findings. The study underscores the necessity for broader and more detailed mechanistic studies to validate these findings and proposes further investigation into the relationship between intra-tumoral and vaginal microbiota, which may contribute to the advancement of cervical cancer prevention and treatment strategies.

## Conclusion

In summary, our investigation has charted the landscape of tumor-associated microbiota in HPVI ECA, uncovering microbial signatures with potential as predictive biomarkers. Utilizing RF algorithms, we’ve linked specific microbiota profiles to clinical outcomes and established their correlation with disease progression. Despite certain limitations, our findings offer a promising gateway to better understand the role of microbiota in HPVI ECA, which may lead to improved diagnostic and therapeutic strategies. As we seek to enhance cervical cancer management, this research lays the groundwork for further exploration into the microbiome’s impact on patient prognosis.

## Data Availability

The datasets generated for this study can be found in the SRA of NCBI: https://www.ncbi.nlm.nih.gov/sra/PRJNA1142715.
