# Supplementary material for: Diagnostic and prognostic potential of the intra-tumoral microbiota profile in HPV-independent endocervical adenocarcinoma
Source: Front Cell Infect Microbiol. 2024 Aug 16;14:1440017. doi: 10.3389/fcimb.2024.1440017 (PMC11362085; doi:10.3389/fcimb.2024.1440017)
Supplement: Supplementary file 1 [file Table1.docx]

Supplementary Material

# Supplementary Tables

Supplementary Table 1. Top 10 microbial compositions by average abundance at Phylum and Genus levels for each group.

Supplementary Table 2. At the phylum and genus levels, microbial differences between different groups were identified using the Wilcoxon rank-sum test.

Supplementary Table 3. The detailed genus composition in each sample.

Supplementary Table 4. Significant differences in KEGG pathways between GEA and CCC groups.

Supplementary Table 5. Significant differences in COG pathways between the Tumor and Para-tumor groups.

# Supplementary Figures

#
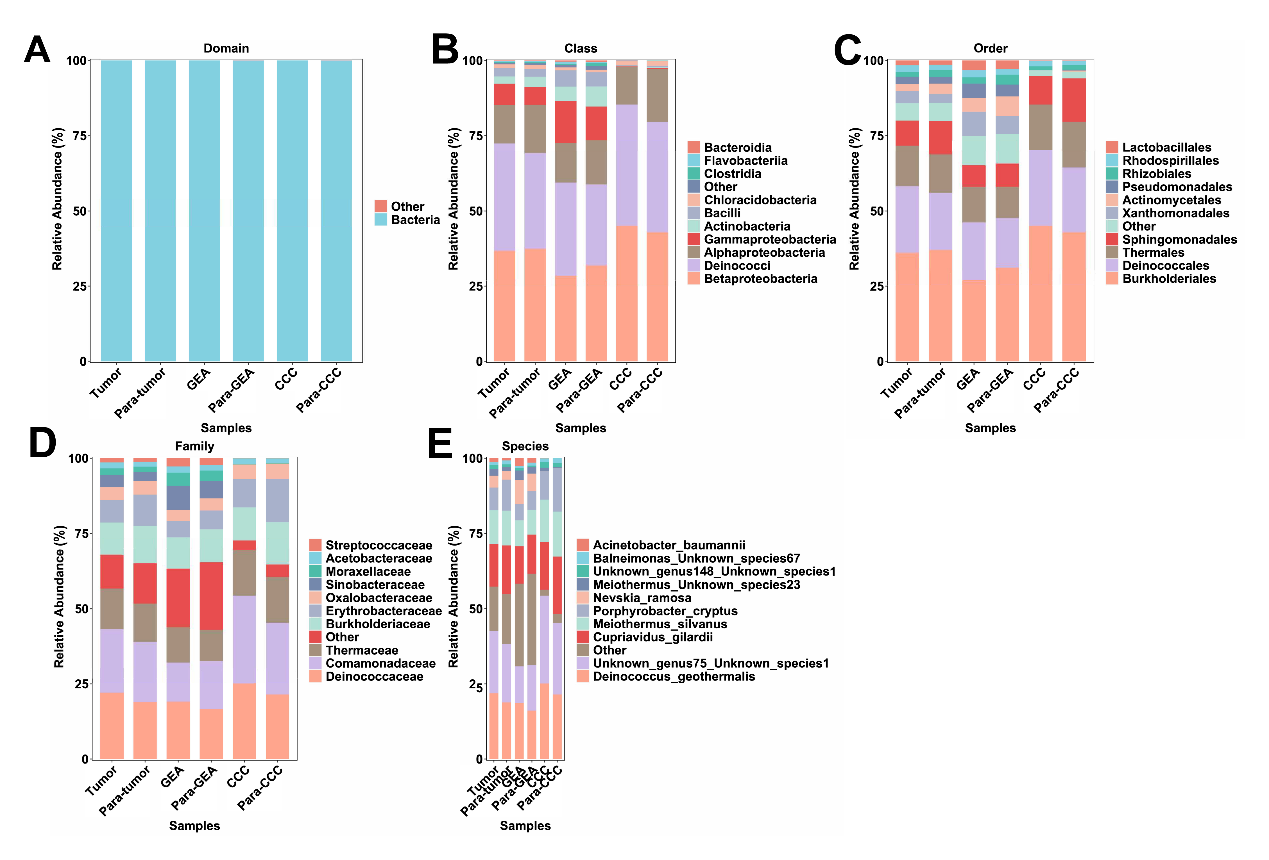


**Supplementary Figure 1.** Stacked bar plot of mean proportions of taxonomic composition in groups at domain (A), class (B) level, order (C) level, family (D) level and species (E) level. (B-E) only displays the top 10 microorganisms.


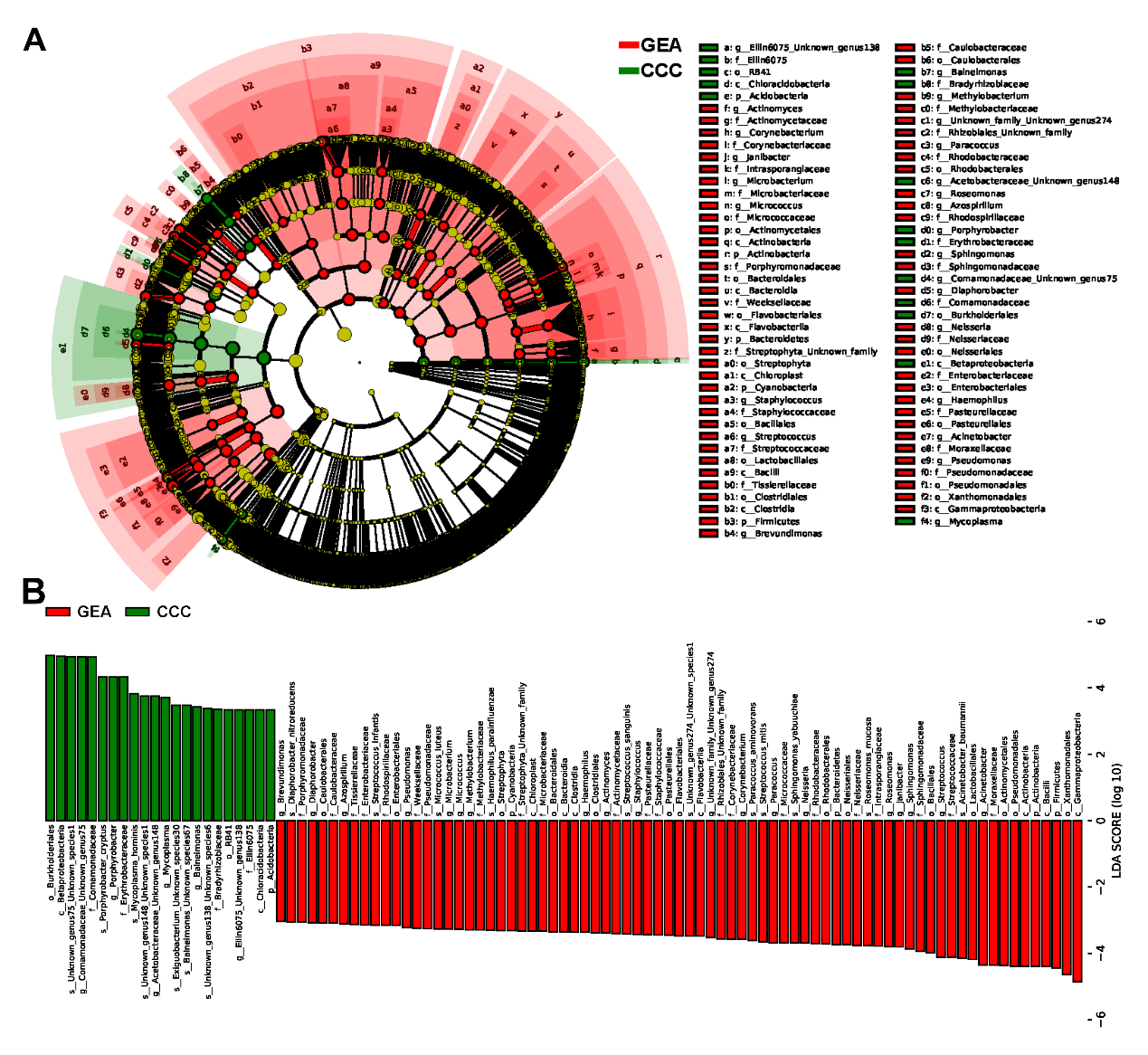


**Supplementary Figure 2.** LEfSe analysis identified dominant taxa in gastric-type endocervical adenocarcinoma (GEA) and clear cell carcinoma (CCC) groups. (A) Cladogram illustrating taxonomic enrichment of microbial taxa between GEA and CCC groups. (B) Linear discriminant analysis (LDA) scores of specific microbial taxa at all levels between GEA and CCC groups.


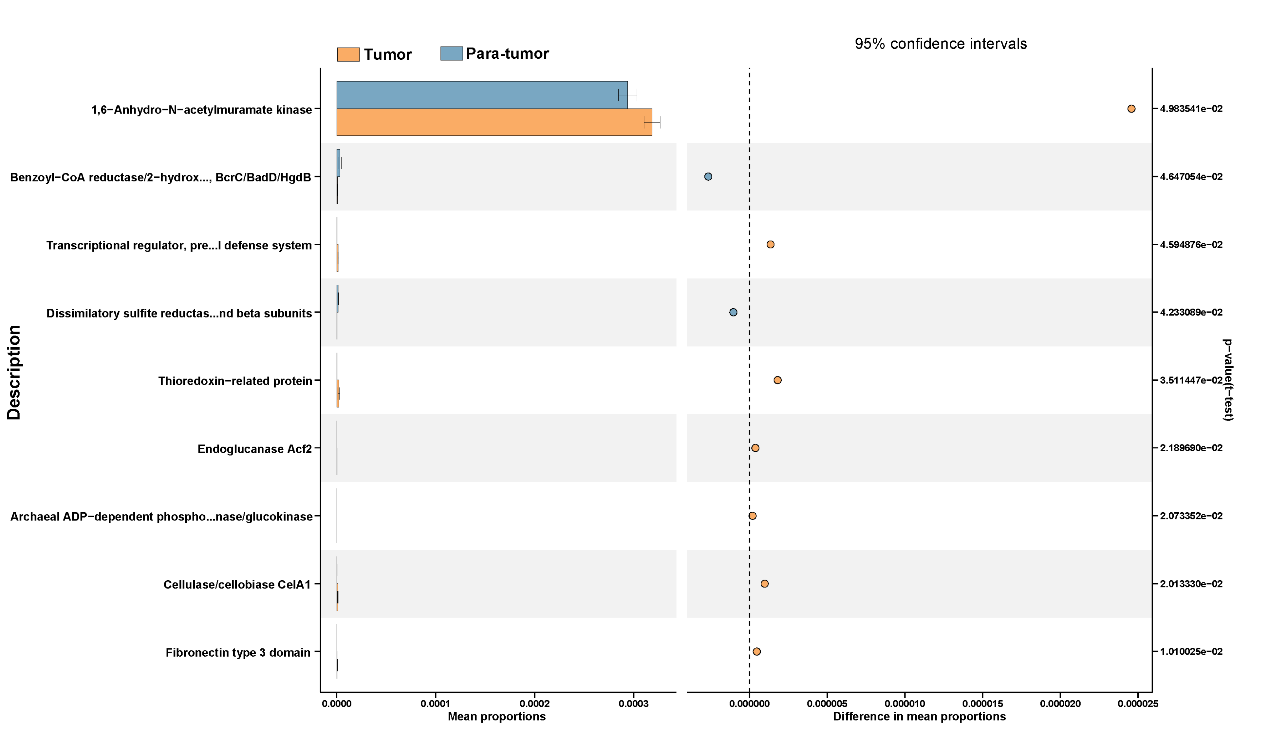


**Supplementary Figure 3.** Nine significant cluster of ortholog genes (COG) functional categories between Tumor and Para-tumor groups.
